# In-vivo measurement of the fluorescence spectrum of wild cochineal (*Dactylopius opuntiae*)

**DOI:** 10.1038/s41598-020-80108-4

**Published:** 2021-01-11

**Authors:** Alejandra Cárdenas Rosales, José Alberto Delgado Atencio, Margarita Cunill Rodríguez, Enrique González Gutiérrez

**Affiliations:** Universidad Politécnica de Tulancingo, Ingenierías 100 Huapalcalco, 43629 Tulancingo de Bravo, Hidalgo Mexico

**Keywords:** Biological fluorescence, Invasive species, Optics and photonics

## Abstract

It is known that the harmful presence of the wild cochineal (*Dactylopius opuntiae*), unlike the fine cochineal (*Dactylopius coccus*), in prickly pear crops of farmers leads to consider it as one of the major pests for this crop. In this study, we present the implementation of an optical setup that ensures the measurement of the in-vivo fluorescence spectra of wild cochineals ranging in size from 440 to 1190 µm in their natural habitat achieved by developing a reproduction model adopted from available literature. It was observed that in-vivo fluorescence spectra of these insects were comprised in the spectral region of 570–760 nm, showing a proportional dependence between the fluorescence intensity emitted and the cochineal size. In addition, we have considered other spectral parameters to perform the comparison between fluorescence spectra of the different cochineal sizes. These results provide the basis for the development of novel methodologies and equipment aimed towards the early detection of this pest in prickly pear crops from its early growth stages (nymph I and II).

## Introduction

The cochineal is an insect that feeds off the prickly pear cactus and from which a red pigment commonly known as carmine is extracted whose use dates back to prehistoric times^1,2^. It is known that the quality of the dye produced by this insect depends on if it is from the fine cochineal or the wild one. The fine cochineal, *Dactylopius coccus*, is distinguished by an external white dusty coating called *coccerin*^3^ while wild cochineal, *Dactylopius opuntiae*, shows an external cottony white cover^4,5^. In contrast with fine cochineal, the wild cochineal does not have a high economic value in food, textile, cosmetics and pharmaceutical industries. On the other hand, its presence in prickly pear and edible cactus farming leads to negative effects to such an extent that it is considered as one of the main insect pests attacking prickly pear crops in several areas of Mexico and other countries^6–11^. Specifically, problematic are the females, who from their first instar, also known as “crawler”, they move on the surface of the plant looking for fresh cladodes to feed. Once the crawler introduces its buccal apparatus into the plant tissue, it will not move again, constantly sucking the sap from the cladode^12^, bringing as a consequence the premature fall of cladodes and fruits before their maturation and eventually the death of the plant^6–10,13,14^.

In general, the farmers notice that their crops are infected by this pest when the cochineal is able to be observed with the naked eye, having at that moment a size of 3.4 mm long by 2.1 mm wide approximately, which according to the study of Rodrigo et al.^15^ is when the insect reaches adulthood. The latter means that at this time the cochineal has already fed, reproduced and deposited eggs on the crop; as a result, it is too late for its control, thus favoring the spread of the pest. Therefore, given the high resolution of optical methods and their intrinsic diversity, they might be excellent candidates for the early and efficient detection of the wild cochineal in cactus crops, thus contributing to timely pest monitoring with the consequent reduction of pesticides and other materials that are currently used in the control and treatment of pests and their effects.

Carminic acid^16^, which is the main chemical component of carmine, has been widely studied by means of optical spectroscopy for chemical applications^17–20^. This component was used in these investigations from commercial products available from companies such as Sigma Aldrich and was always diluted in solvents such as water, methanol, boron and hydrochloric acid. In these studies, research on different issues of the fluorescence of this compound is reported.

However, carminic acid is found also naturally within the cochineal as a part of the hemolymph of this insect^3,15,21^. Its concentration has been studied depending on different factors. For example, Rodríguez et al.^22^ showed that the weight of this insect and the age of the cladode on which they feed cause variations in the carminic acid concentration present in ex-vivo cochineal. Later Flores et al.^21^ studied the variation in the concentration of carminic acid with the different maturation stages of ex-vivo cochineal through capillary electrophoresis and flow cytometry in the measurement of the total fluorescence of the carminic acid globules of this insect. In contrast to these two studies, in a recent investigation, Cárdenas^23^ reported the observation of in-vivo fluorescent microscopic images of wild cochineal. This result was obtained when a laser pointer emitting at 532 nm was used as excitation source, while in-vivo fluorescence observation was performed by placing a band pass filter (10BPF10-660, Newport Corporation) in one of the ocular tubes of the microscope (PST-24-10L, Parco Scientific) to which a color CMOS camera (FL3-U3-13S2C-CS, FLIR Integrated Imaging Solutions, Inc.) was attached^23^.

To the best of our knowledge, there are no existing research articles that report in-vivo measurement of the fluorescence spectrum of cochineal (fine or wild). Therefore, the detailed investigation of in-vivo cochineal fluorescence microscopy and spectroscopy would contribute to a better comprehension of the scope of the fluorescence of this insect as a basic principle for its early detection in the prickly pear cactus fields.

In this research an optical setup that ensures measuring the in-vivo fluorescence spectrum of wild cochineal in its natural habitat is reported. The optical setup essentially consists of a miniature fiber optic spectrometer, a dichroic mirror and two microscope objectives, one of which ensures the “punctual” excitation over the cochineal and the other concentrates the fluorescent radiation in the optical fiber connected to said spectral device. Additionally, the dependence of the fluorescence spectrum of this insect with its size in the early developmental stages is also studied. It was possible to measure the fluorescence spectrum of wild cochineals sized between 440 and 1190 µm, observing that the maximum emission wavelength oscillates in the range of 620–667 nm owing possibly to the movement generated by the stimulation of the excitation light over the live-female cochineal, while the spectral emission band was practically the same for all sizes (570–760 nm). In addition, it was found that in this band, the fluorescent spectral intensity level rises with the increase in the size of the cochineal. We proposed and tested a simple and inexpensive but useful fluorescence standard for adjusting the optical setup and optimizing the fluorescence detected signals previously to the spectra recording process.

## Results

**Figure 1:**
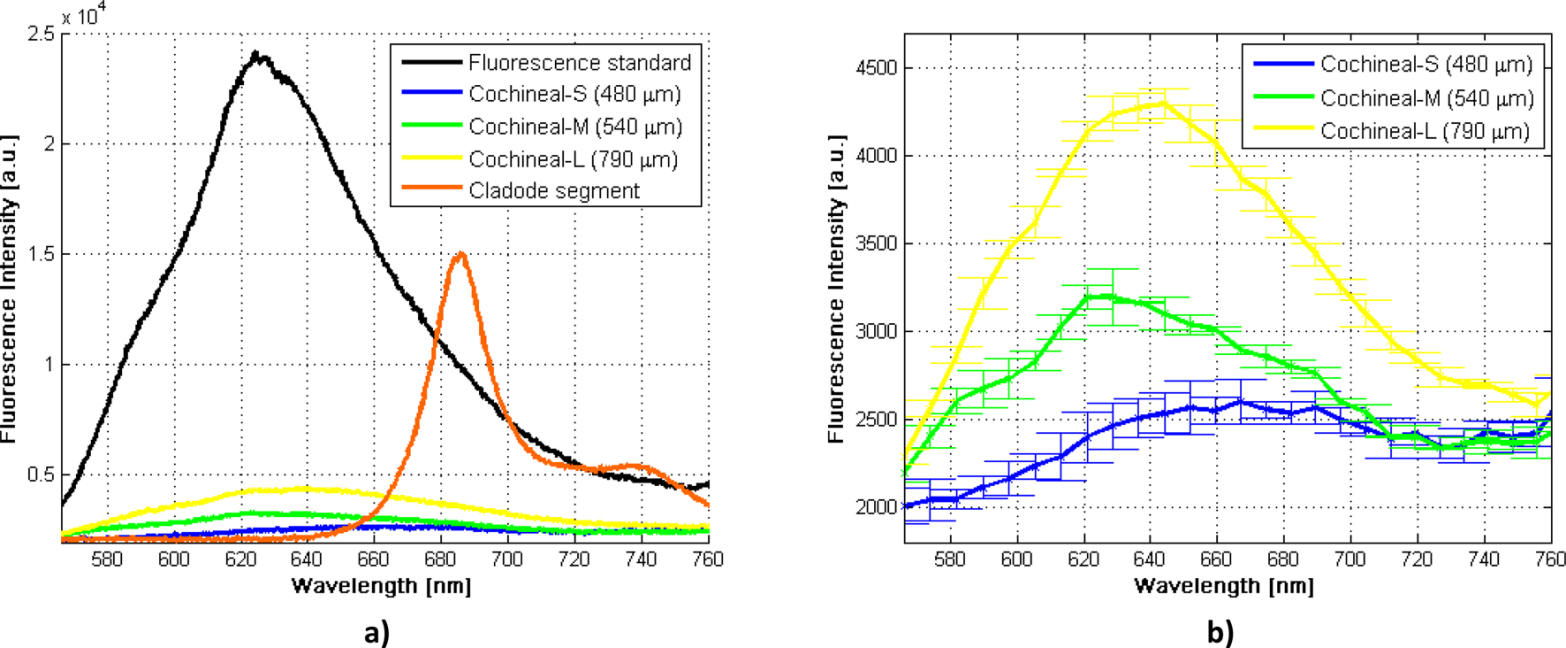
Fluorescence spectra measured with the optical setup presented in this work. (**a**) Comparison between the fluorescence spectrum of the carmine colored pencil standard, the in-vivo cochineals fluorescence (small, medium and large) and in-vivo cladode segment fluorescence. (**b**) In-vivo fluorescence spectra of wild cochineals of different sizes.

The spectral data acquired from the samples are plotted as shown in Fig. 1a. In this figure, the fluorescence spectra of the carmine colored pencil standard, the in-vivo fluorescence of small, medium and large cochineal and finally, the fluorescence of the cladode segment are illustrated.

It is noticeable that each spectrum shown in Fig. 1a keeps different fluorescence intensity; the fluorescence spectrum of the carmine colored pencil standard having the highest emission, followed by the fluorescence of the cladode portion and finally with significantly less intensity the spectra of the small, medium and large cochineal, respectively.

In order to distinguish details such as the shape and the difference of fluorescence intensity between the three spectra, corresponding to the different sized cochineals, only these 3 spectra were plotted in Fig. 1b. In addition to being averaged and smoothed, observe that the standard deviation was computed and plotted for each spectrum. As shown in this figure, the fluorescence emission spectral bands were detected within the visible region of the electromagnetic spectrum (570–760 nm), showing maximum emission peaks at different wavelengths, 666, 621 and 644 nm for the small, medium and large cochineal, respectively. These results allow us to confirm the existence of in-vivo fluorescence of the wild cochineals as a result of the presence of carminic acid located inside them. As observed in Fig. 1b, the fluorescence intensity of each spectrum is proportional to the size of the cochineal; that is, the small cochineal emits lower fluorescence while the large cochineal emits higher intensity.

**Figure 2:**
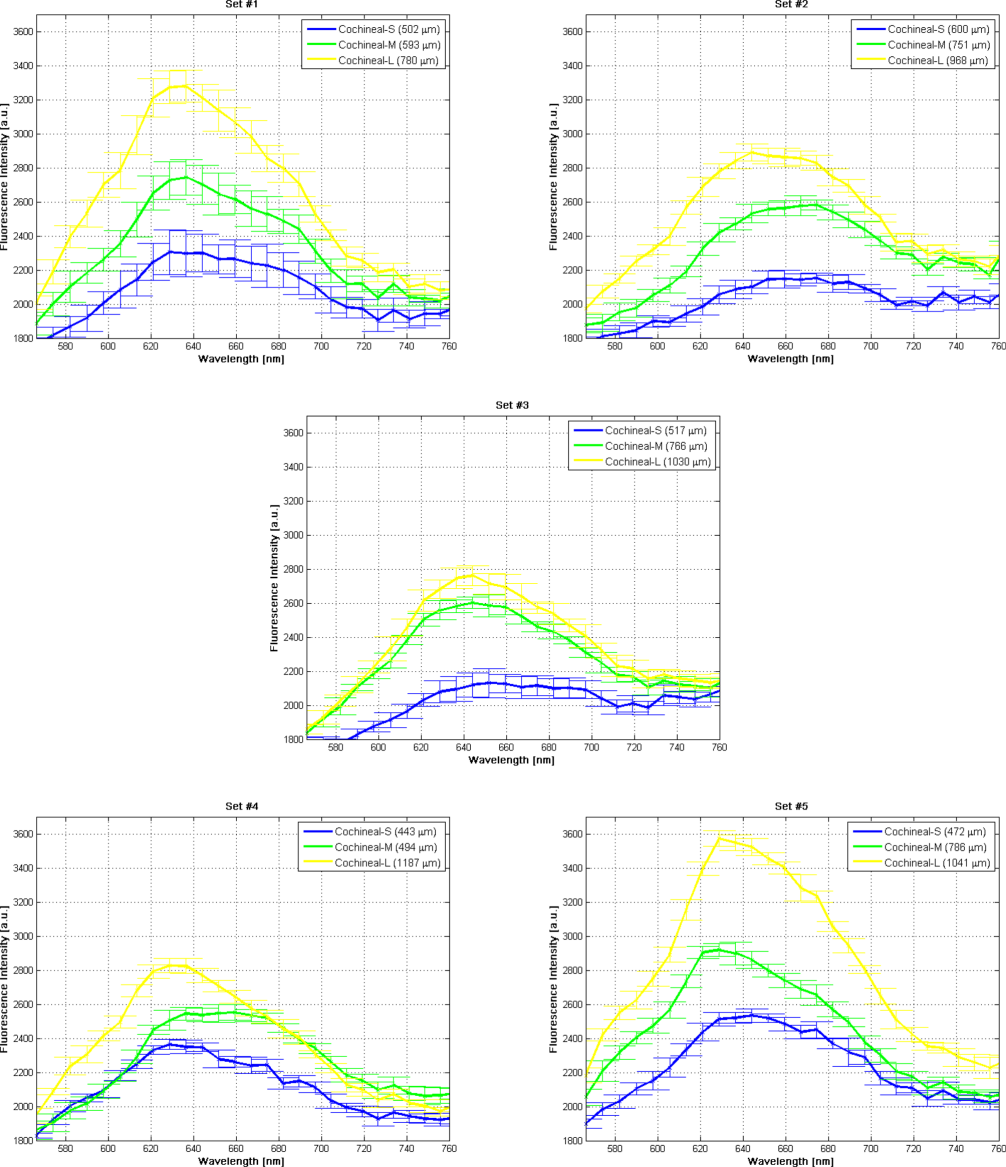
Graphical comparison of in-vivo fluorescence spectra of wild cochineals of different sizes for the five sets of biological samples described in section "Materials and methods”.

In order to confirm the reliability of our results in this proof of concept and for statistical purposes, we have repeated these measurements in five additional sets of cochineals of similar sizes to those above reported. As it is shown in Fig. 2, the spectra for the five sets of the biological samples studied here exhibit a similar spectral shape in the 570–760 nm spectral region. In addition, we found that the higher the size the cochineal the higher is the intensity of the in-vivo fluorescence spectrum around the band of maximum emission. In Table 1 the approximate sizes in microns of the cochineals studied in each repetition are shown.

**Table 1:** Approximate cochineal sizes in microns used in each additional measurement of in-vivo fluorescence.

Cochineal size (µm)	Set 1	Set 2	Set 3	Set 4	Set 5
Small	502	600	517	443	472
Medium	593	751	766	494	786
Large	780	968	1030	1187	1041

It is observed from Table 1 that the size of the cochineals ranges from 440 to 600 µm, 490 to 790 µm and 780 to 1190 µm for the small, the medium, and the large cochineal, respectively. It was possible to verify that for these additional sets a similar behavior as the one reported in Fig. 1b was observed as shown in Fig. 2, even when the size range between the small and medium cochineal was overlapped.

In the case of the analysis of the spectral parameters for comparing fluorescence spectra, we found a similar behavior for sets 1, 4 and 5 with comparable bandwidth values very close to each other, while for sets 2 and 3 the bandwidth values were different showing a minimum value for the small cochineal as well as the same bandwidth for the medium and large cochineals in both cases, as shown in Table 2. Note that for sets 1, 3 and 5 the maximum emission wavelengths shown a similar conduct, where medium and large cochineal had the same emission wavelength, as well as set 2 for small and medium cochineal and set 4 for small and big cochineal.

**Table 2:** Spectral parameters obtained of each set of in-vivo fluorescence spectra as shown in Fig. 2, when processing in a script development in MATLAB R2014a (The MathWorks, Inc.) software.

Set	Spectral bandwidth (nm)			Maximum emission wavelength (nm)			Maximum fluorescence intensity (a. u.)		
	S	M	L	S	M	L	S	M	L
1	158	159	159	628.71	636.42	636.42	2305.6	2743.8	3277.4
2	160	189	189	674.50	674.50	644.09	2151.9	2581.2	2890.3
3	145	189	189	651.74	644.09	644.09	2130.0	2601.4	2760.5
4	189	182	189	628.71	659.35	628.71	2363.4	2550.9	2827.0
5	189	189	189	644.09	628.71	628.71	2533.4	2918.9	3572.2

Note that the values of the spectral bandwidth, maximum emission wavelength and maximum intensity are grouped by each set of cochineals, classified by the sizes presented in this study as S, M and L.

This study allowed us to determine maximum emission wavelength ranges to each repeatability set: 628.71–636.42 nm for set 1, 644.09–674.50 nm for set 2, 644.09–651.74 nm for set 3, 628.71–659.35 nm for set 4 and finally 628.71–644.09 nm for set 5.

**Figure 3:**
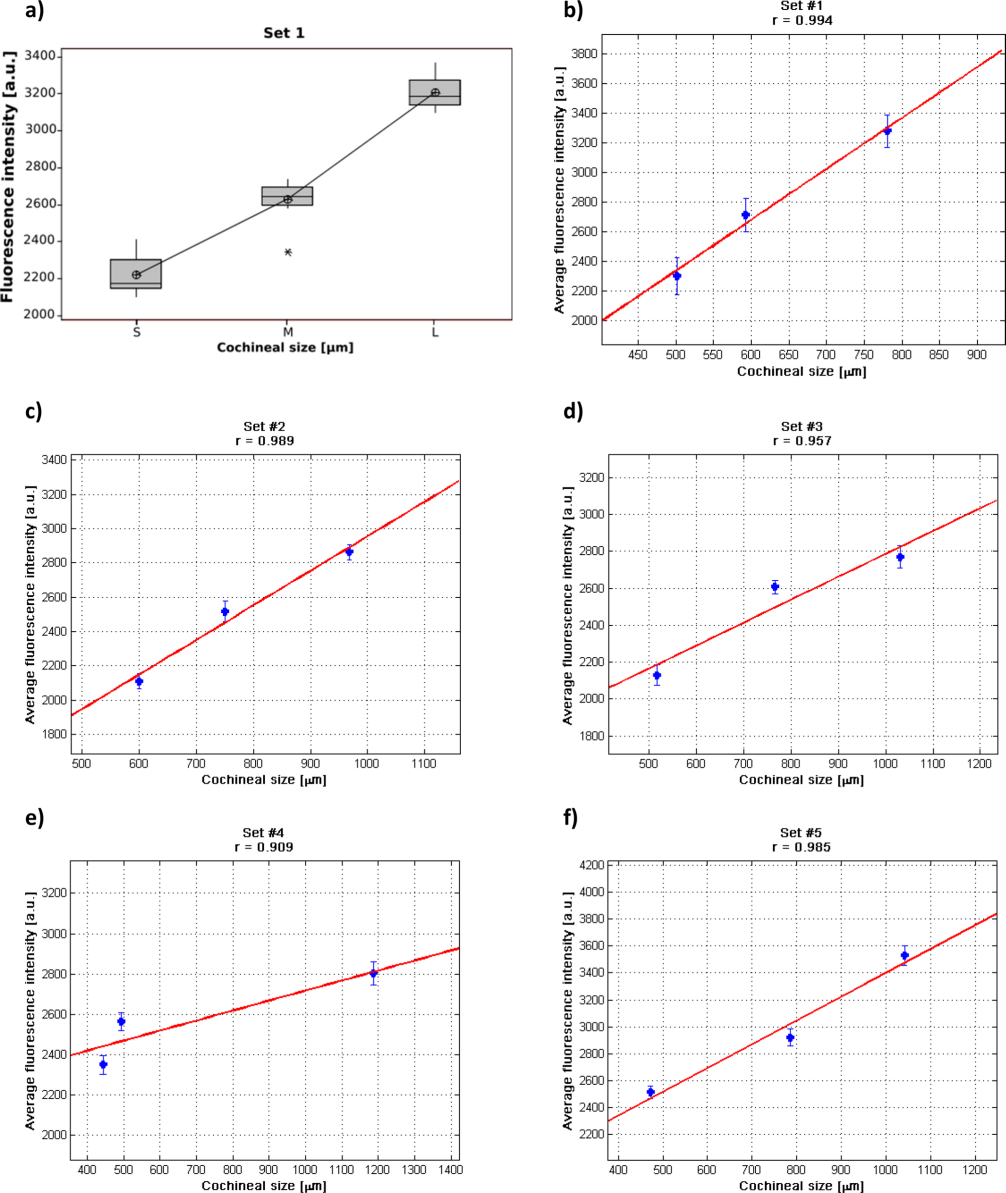
Average fluorescence intensity as function of cochineal size. (**a**) Typical box plot for the set #1 at 620 nm using ANOVA method. (**b**–**f**) Linear fitting (red line) applied to different repeatability sets 1 to 5, respectively, at the wavelength of 640 nm for each data set (blue dots) using MATLAB, a significant difference in the processed spectra shown in Fig. 2 is noticeable.

Figure 3 presents the average fluorescence intensity as function of cochineal size. In Fig. 3a, a typical box plot of the average fluorescent intensity at 620 nm is shown, meanwhile in Fig. 3b–f a typical behavior of the dependence of fluorescence intensity at 640 nm is observed.

As can be seen in Fig. 3, the same behavior is observed for the 6 plots: the fluorescence intensity increments as the cochineal size increases. From the spectral analysis of each set at the wavelengths 620, 640, 660 and 760 nm, and from ANOVA analysis processed in Minitab 16 (Minitab, LLC.) software, we concluded that the different cochineal size (small, medium and large) was always statistically significant for the measured intensity of fluorescence at all these wavelengths, as shown in Fig. 3a at 620 nm. The results obtained of the spectral data (blue dots) were fitted (red line) as mentioned in Method section for the five biological sample sets and are presented in Fig. 3b–f, respectively. As it is observed from these figures, a good linear relationship was obtained between the average fluorescence intensity and the cochineal size at the emission wavelength of 640 nm. A similar behavior was obtained when the analysis was performed at 620 and 660 nm.

On the other hand, we have proposed the carmine colored pencil as the fluorescence standard in this study, due to the absence of a commercial one in our laboratory. We measured 10 fluorescence spectra of the carminic acid standard to study its stability over time (data no shown). The temporal stability obtained by these measurements as well as the nearness of its maximum peak (624 nm) with the peaks of fluorescence emission of the cochineals, allows us to suggest using this carmine colored pencil as a standard for in-vivo fluorescence measurements of cochineals in our experimental setup. In this scenario, such fluorescence standard is mainly used during the aligning step of the optical set up to maximize the fluorescence signal recorded at the spectrometer. Besides, this standard is easy to use for comparison purposes.

**Figure 4:**
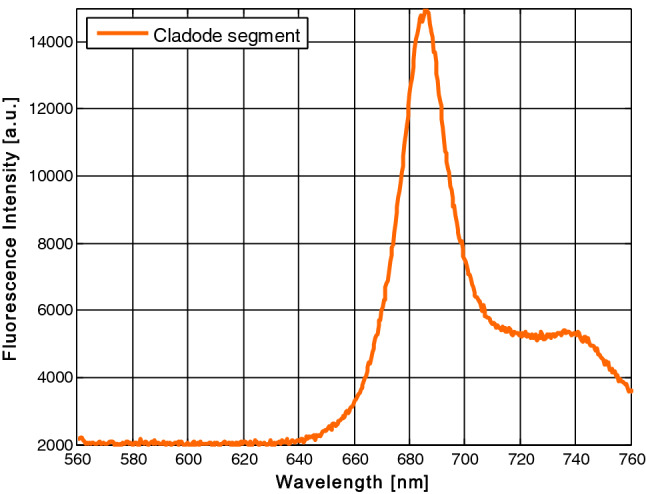
Fluorescence emission spectrum of the cladode portion on which the different cochineals were placed for spectra measurements in Fig. 1a.

Figure 4 shows the fluorescence spectrum of the cladode surface where the cochineals were placed. This spectrum shows a maximum emission fluorescence peak at 686 nm, and this is extremely high in intensity in comparison with the fluorescence spectra of cochineals as can be observed directly from Fig. 1a. This spectrum was recorded when the excitation laser beam was focused on the cladode surface. It is important to notice that there is a red shift of peak intensity of this spectrum of about 20 nm with respect to the in-vivo fluorescence peak intensity of the small cochineal (666 nm).

Lastly, but not less important, we have ensured the availability of cochineals to perform measurements at different time periods through a cochineal reproduction model fundamentally based on the infection of healthy cladodes through points of contact with a cladode already infected with the pest. This method allows us to keep this pest in constant development supported by the fact that, as observed in our research, an infected cladode with the pest on both sides is enough to contaminate 4 to 5 healthy adult cladodes. By having cladodes planted in pots, this method is manageable and allows them to be exposed to sunlight for the proper growth of the pest according to its life cycle and be returned to the laboratory to perform the fluorescence measurements at a temperature range from 20 to 25 °C as well as a relative humidity range from 42 to 80% during a time of 30 min for each cochineal set, as observed on Table 3. Additionally, being housed in the laboratory protects them from possible predators outside as well as climatic factors such as the wind and the rain, thus maintaining appropriate care and conservation of the pest. In this study, a photoperiod of 9 ± 1 h of direct solar radiation was stablished during 3 months from 09:00 to 18:00 h at last five days of life cycle of the cochineal, observing a maximum solar radiation between 13:00 and 14:20 h with a maximum solar radiation of 1037 to 1322 W/m^2^. These climatic parameters (temperature, humidity and solar radiation) were obtained from the UPT weather station (Vantage Pro2, Davis Instruments Corp.).

**Table 3:** Temperature and humidity registered on online and the UPT weather station from cochineal life cycle (photoperiod) stages until the experimental fluorescence measurements.

Stage	Set	Date	Temperature		Humidity	
			Web (°C)	UPT weather station (°C)	Web (%)	UPT weather station (%)
Life cycle of cochineal (photoperiod)	–	March 5th	15.5	–	65	–
		April 5th	20.7	–	49	–
		May 5th	16.5	–	75	–
		June 5th	22.5	26.40	60	35.5
Rest	–	June 6th–24th	21.5	21.22	68	59.78
Main measurement	0	June 19th	19	24.35	75	43.5
Measurement (replicas)	1	June 25th	19	23.71	84	54.92
	2	July 5th	18	21.88	74	64.42
	3	July 11th	19	18.78	85	79.21
	4	July 23rd	18	21.33	78	66.78
	5	July 26th	19	20.29	77	67.85

## Discussion

An optical setup through which was demonstrated the existence of in-vivo fluorescence spectrum in different sized wild cochineals (*Dactylopius opuntiae*) in their natural habitat was presented in this work. The main objective of the paper was a proof of concept for measuring the above-mentioned spectrum of wild cochineals ranging from small to large sizes (440–1190 µm).

We found that for a measurement set composed by three live-female cochineals of sizes ranging between 440 and 1190 µm, the emission fluorescence spectra of each insect show different peaks of maximum intensity in 666, 621 and 644 nm for the cochineals classified as small, medium and large, respectively. We believe this difference in the position of maximum fluorescence emission peaks, could have its origin due to the experiment being in-vivo and these organisms, although attached to the cactus, could have some freedom of movement^4,23^ during the acquisition time of the spectrum. Also, if we consider that laser power density focused on the cochineals could be relatively high (~ 1.119 W/cm^2^), which could produce certain excessive heating on the cochineal surface and volume generating discomfort and causing them to move in order to avoid such stimulus. Therefore, the excitation light spot could randomly strike on the dorsal structure of the cochineal and generate a fluorescence spectrum which may vary during the typical integration time used in this study (200 ms), resulting in a range of spectra to be defined for each sample measured. We consider these small variations of spectra, due to the cochineal movement, by performing 10 measurements of the fluorescence spectrum for a single cochineal and reporting its average value and standard deviation. For statistical purposes, we have repeated these measurements 5 times more by using cochineals sets of similar sizes to those reported in this work, as shown in Fig. 10. As a result of this study of repeatability, we have observed a similar proportionality behavior between the intensity of fluorescence intensity spectra and the size of each cochineal within each set as grouped in Table 1.

It is important to highlight that the dorsal form of the cochineal is almost similar to an ellipsoid of revolution cap with superficial waves parallel to each other^4,5,23,30,31^. During the movement of the cochineal, due to the stimulus generated by the laser focus, the excitation light spot can strike on different sections of the surface and this could lead to differences in the fluorescence signal collected by the microscope objective. The collected signal may depend on the light spot-objective distance, the angle of incidence with respect to the normal of the dorsal surface, among other factors. In this sense, it was found that the intensity level of each spectral band of fluorescence emission rises as the size of the studied cochineal increases. Essentially, the method used for comparing fluorescence spectra in this work consisted on a graphical testing based on the spectral parameters (see Table 2), which allowed us to observe that all spectra tend to have the same bandwidth, and emission wavelengths of each cochineal are very close to each other. The scope of this work demonstrates the difference between spectra is notable due to the fluorescence intensity of each experimentally measured set of cochineals of different sizes, as well as the other spectral parameters could be related to cochineal heating, which can be addressed in future research.

During the lifecycle in the nymph I and II stages, our results are not inconsistent with those reported by Flores et al.^21^, where they found that there was an increase of total fluorescence of carminic acid of ex-vivo samples of fine cochineal due to the increase of carminic acid concentration with different growth stages. We have observed a similar behavior^21^ in our research study for in-vivo wild cochineal samples, where the fluorescence emitted by cochineals in nymph I and II stages increases with cochineal size. Despite the relationship reported by Flores et al.^21^, we have considered that in our studies the growing fluorescence intensity between the cochineals is directly related to the insect size and not to the carminic acid concentration inside this insect. In order to support this sentence, a linear fitting of the average fluorescence intensity as a function of the cochineal size was performed at four selected wavelengths at 620 nm, 640 nm, 660 nm and 760 nm (see Fig. 3b–f), which allow us to verify that fluorescence intensity as a function of the cochineal size embracing a linear model fitting. Also, we computed the correlation coefficients from each set presented above, where in most of the cases these coefficients were very close to 1 in a range of 0.919–0.999 at 620 nm, 0.909–0.994 at 640 nm, 0.718–0.990 at 660 nm and − 0.101 to 0.930 at 760, showing a positive correlation except when the coefficient is equal to − 0.101 that results when the spectra are difficult to distinguish. This important analysis supports the main finding of this work: *the fluorescence intensity is proportionally constant to the size of cochineal*.

Our findings are more general than reported by Cárdenas^23^, since we confirm that the origin of the fluorescent microscopic images reported by this author could take place even in the habitat of the cochineals and not only over an artificial substrate as it is stated in that research work. Moreover, the methodology of our work exceeds that used by Cárdenas^23^, since the dichroic mirror of our optical setup guarantees that the emission band of the carminic acid contributes in full to the spectral signal recorded while the bandpass filter used in the optical setup of that research to obtain fluorescent microscopic images was not ideal owing to logistical limitations.

We also consider relevant to highlight here, that a cylindrical segment of a carmine colored pencil was proposed and tested as a fluorescence standard for this optical setup, due to the absence of a commercial one in our laboratory^32^. This proposal is mainly based on the fact that, although its emission spectrum is considerably higher than the cochineals studied (see Fig. 1a), the spectrum shape is very similar to the fluorescence spectrum of the carminic acid diluted in different liquid solutions^18,19,23^ as well as the emission spectral profile of the cochineals measured in this study. A maximum intensity peak at 624 nm is noticeable for this fluorescence standard, near the emission peak of the medium cochineal (621 nm). This standard showed acceptable spectral intensity stability during fluorescence measurements. Therefore, we propose that it could be used as a fluorescence standard of carminic acid for future studies related to this topic.

It is relevant to mention that, as far as we know, this is the first study which investigates and specifies the spectral band of in-vivo fluorescence emission of the wild cochineal in its natural habitat and compares it with the fluorescence spectrum of cladode. In this study we have demonstrated the existence of in-vivo fluorescence of the cladode through the fluorescence emission spectrum of the portion of the cladode in which the cochineals of different sizes were parasitizing. As was appreciated in Fig. 2, the emission band shape of the cladode spectrum as well as the maximum emission intensity peak around 686 nm, are similar to the typical fluorescence spectrum of the chlorophyll extracted from spinach leaves dissolved in alcohol as reported by Murphy and Davidson^24^ when detected fluorescence emission of this solution at 670 nm by its excitation at 420 nm. Similarly, Cerovic et al.^25^ reported that chlorophyll dissolved in methanol has its fluorescence emission peak approximately at 685 nm when it was excited at a wavelength of 450 nm. Finally, another similar result is shown on the website of Prahl and Jacques^26^ about the research of Weber^27^ where the chlorophyll dissolved in diethyl ether has a fluorescence emission peak at 666 nm when it was excited at a wavelength of 614 nm.

Dissimilar to our finding, Murphy and Davidson^24^ mention that “Normally, the chlorophyll and other pigments in intact chloroplasts act as energy transducers and do not fluoresce”, which is not observed in our results. For this reason, in this study the existence of the in-vivo chlorophyll fluorescence is also demonstrated by means of the emission spectrum of the cladode portion presented in Fig. 4. This finding could be explained if we assume that perhaps a certain fraction of chlorophyll exists outside the chloroplasts and is dissolved in some of the liquid elements that compose this tissue.

Despite the maximum intensity of fluorescence emission of the cladode at 686 nm, being 3.5 times higher than the corresponding fluorescence emission of the large cochineal at 644 nm, the inclusion of an adequate band pass filter in a future field instrument, allowing only an emission band centered in this last wavelength to pass, must serve to visualize the desired in-vivo fluorescence of cochineal. This approach could become in the main element for the early detection of cochineal pest in crops by farmers. Therefore, it is proposed to use a commercial filter (MF630-69, Thorlabs Inc.) with central wavelength in 630 nm which adequately blocks both the excitation wavelength and the fluorescence emission wavelength of the cladode portion. Particularly in this study, it turns out to be of great importance to include this kind of optical device to discriminate the unwanted wavelengths and to carry out the in-vivo detection of the wild cochineal through fluorescence techniques with the aim of being part of the instrumentation that is going to be used in the crops by the farmers and the specialized staff. The transfer of this technology to adequate conditions such as those of a prickly pear field could be implemented by a portable optoelectronic device based on the results reported in this research work. Essentially, the prototype could include the use of a special optical system, a ring of LEDs that provides the excitation beam at the appropriate wavelength, and the display of cochineals by visual and/or assisted detection by a two-dimensional sensor coupled to an LCD mini-screen. This device will provide a useful tool for farmers all over the world that face the problem of early detection of this that affect prickly pear fields.

The research we have presented in this study, provides basic elements that support the working principle of a novel method aimed at the in-vivo detection of the wild cochineal pest by incorporating fluorescence techniques, in order to design and develop field instruments that help to monitor and determine the presence of this kind of pest in its early growth stage in prickly pear crops. Hence, it contributes to the reduction of chemical pesticides used in the fighting of this pest, by detecting the cochineal when it has not developed its white waxy coating on the outside of its body that protects it from predators and provides resistance at chemical pesticides^33,34^, such as malathion, parathion, among others^33–35^. Therefore, a consequent economic savings for prickly pear farmers and/or prickly pear producers of the state of Hidalgo and other producing regions of this crop can be achieved. As well as applications of this technique worldwide, where the existence of the problem with the pest of the cochineal is known.

## Materials and methods

In this section, the methodology for the fluorescence spectra acquisition of the in-vivo cochineal in its natural ambient is described. The optical setup used is our own design that guarantees the detection of low levels of fluorescence present in this study. In addition, the wild cochineal reproduction model employed to ensure the existence of the samples is described. This study proposes a fluorescence standard based on a commonly used disk-shaped carmine colored pencil segment because of the non-availability of a commercial one in our laboratory.

### Optical setup for fluorescence spectra measurements

**Figure 5:**
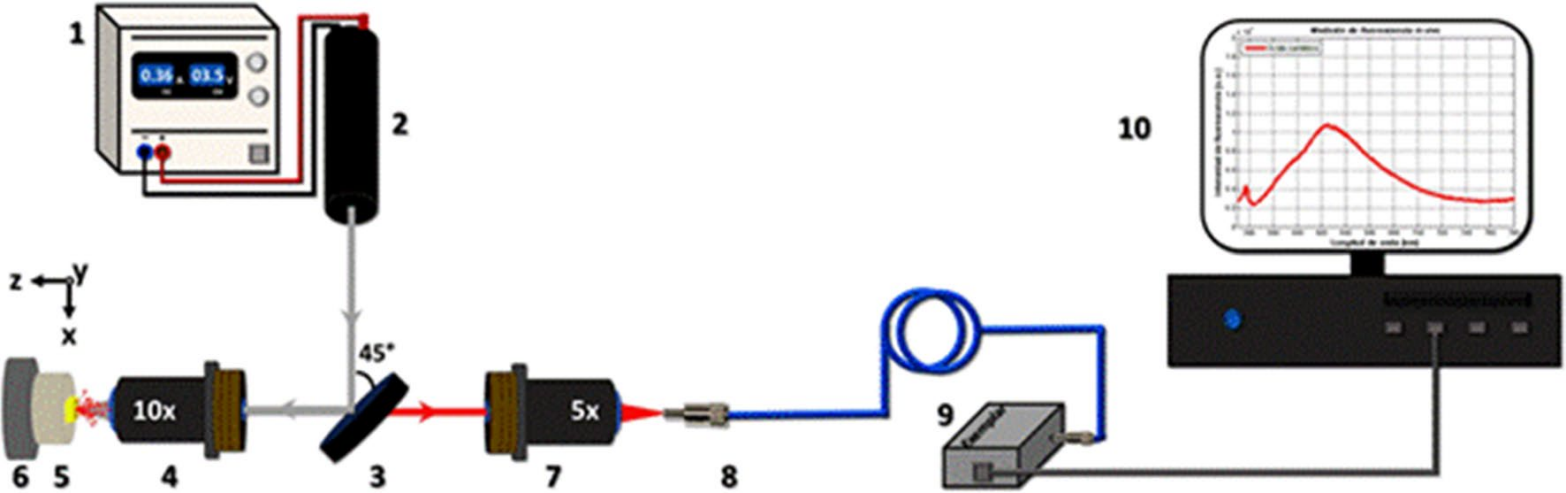
Optical experimental setup for detecting fluorescence spectra: (1) power supply, (2) laser source emitting at 532 nm, (3) dichroic mirror, (4) 10 × microscope objective, (5) sample-holder with the biological specimen depicted in yellow color, (6) a mechanical positioning device with micrometric movements in *x*, *y* and *z* directions, (7) 5 × microscope objective, (8) optical fiber, (9) fiber optic spectrometer and (10) desktop computer showing a typical spectrum of carminic acid within a cochineal.

The optical setup for detecting fluorescence spectra consists of (1) a power supply, (2) an excitation source, (3) a dichroic mirror, (4) a 10 × microscope objective lens, (5) a homemade sample-holder, (6) a mechanical positioning device with micrometric movements in *x*, *y* and *z*, (7) a 5 × microscope objective lens, (8) multi-mode optical fiber, (9) a miniature fiber optic spectrometer and (10) computer equipment. The previous elements were arranged as shown in Fig. 5.

The power supply (M30-TP305E, Shanghai MCP Corp.) provides a constant voltage of 3.5 V to the excitation source. The excitation source is a commercial laser pointer (KG-303-6) emitting at a wavelength of 532 nm and nominal power of 1 W. This wavelength is found within the absorption band of carminic acid diluted in methanol^19^, and as was demonstrated by Cárdenas^23^, this wavelength allows the excitation of the fluorescence of natural carminic acid from wild cochineal in-vivo. The dichroic mirror (DMLP550, Thorlabs, Inc.) is tilted at an angle of 45° with respect to the direction of laser beam propagation allowing the excitation light to be reflected towards the aperture of the 10 × microscope objective lens (M-10X, Newport, Co.) which in turn focuses this radiation on a plane where the sample is placed. The specimen to be studied is fixed on the sample holder which in turn is magnetically coupled to the mechanical positioning device (M-900, Newport, Co.), that together with a micrometric base (High-Performance Linear Stage, Newport, Co.) allows the cochineal to be placed at the focal point of the excitation beam. Both the fraction of the fluorescent light emitted isotropically by the cochineal and the fraction of the light reflected diffusely by this insect enter the 10 × objective lens and leave it as a collimated beam towards the dichroic mirror. The first fraction of light is transmitted approximately 85% to the second microscope objective lens (M-5X, Newport, Co.) while the second one is strongly blocked by the dichroic mirror. A 400 µm diameter optical fiber (QP400-2-SR, Ocean Optics, Inc.) is placed at the focal distance from this latter microscope objective to collect the fluorescence radiation and transmit it to the input port of the miniature fiber optic spectrometer (Exemplar, B&W Tek, LLC.) which allows for the spectral decomposition of the fluorescence radiation for its later processing. This spectrometer is connected through a USB port to a desktop computer where the BWSpec (B&W Tek, LLC.) software is installed, provided by the same manufacturer of this device, by means of which the acquisition of the spectra is carried out with an integration time of 200 ms. This software allows the user to save the spectra in plain text files (.txt) for further processing of the acquired information.

The use of the dichroic mirror in the optical setup allows for adequate discrimination of the excitation and emission wavelengths. It shows about a 90% reflection in the range of 380–535 nm, the range in which the wavelength of the excitation beam is found (λexc = 532 nm), whereas for wavelengths in the range of 565–800 nm it presents an 85% transmission. We expect that the in-vivo cochineal fluorescence emission should be mainly located in this last spectral region, based on the findings reported in previous research works for solutions of carminic acid in methanol^19,20,23^.

### Carminic acid fluorescence standard

As a part of this research, we proposed the use of a segment from a carmine colored pencil (Ekuz Carmín, AZOR) as a fluorescence standard due to the absence of a commercial one in our laboratory. This homemade fluorescence standard was used to calibrate the spectra acquisition optical setup. The standard was obtained by cutting a 5 mm segment of the carmine colored pencil with a razor blade (Stainless blade, Dorco, Co.) to achieve a finer cut. Then, this segment was situated inside a plastic piece in an annulus-shape to provide protection and handling during fluorescence measurements. Finally, its face of greater diameter was polished with fine grit sandpaper (B-99 1000, Fandeli, México) to get a smooth plane surface. The opposite face was adhered to the sample-holder while the polished one was placed perpendicular to the optical axis of the 10 × objective lens so that the excitation light impinges on it allowing the optimization of the fluorescence signal recorded by the optical setup.

In order to test the temporal stability of the carminic acid fluorescence standard, a continuous acquisition of fluorescence spectra was performed by using the optical setup previously shown in Fig. 5. To do so, the excitation light source was turned on 30 min before the measurements. Then, the spectra acquisition was done one after the other by allowing the excitation light hits on the standard to detect its fluorescence, immediately blocking this radiation while its spectrum was recorded, and so on until 10 spectra were recorded.

### Biological samples and reproduction model

The cochineal samples used in this research come from a cladode infected with this pest donated by farmers from the Sociedad Cooperativa Productora Agropecuaria de Nopal Tlanalapa S.C. de R. L. de C.V., from the state of Hidalgo, Mexico.

**Figure 6:**
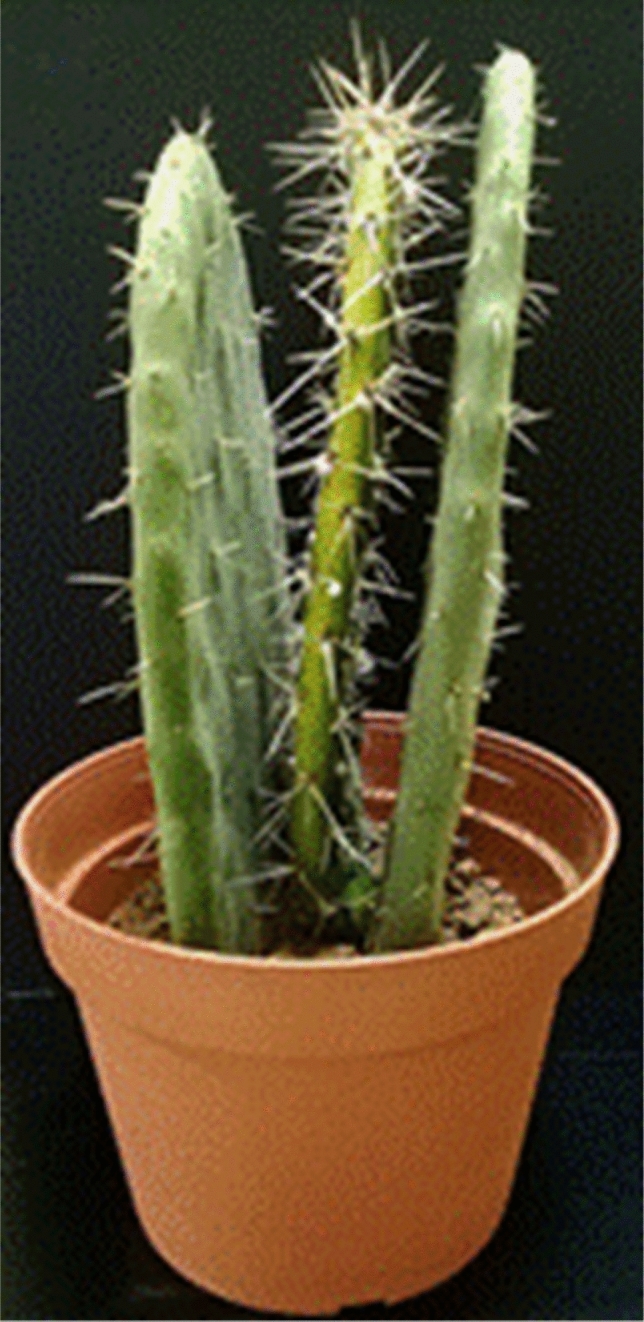
Reproduction model of the cochineal pest in a pot with adult cladodes near an infected cladode with active wild cochineal. Observe, in the center of the pot and between the two healthy cladodes, the infected cladode.

For its survival, this pest was reproduced in the Biomedical Optics Laboratory of the Polytechnic University of Tulancingo, according to the reproduction model illustrated in Fig. 6, in a small-implemented orchard of plastic pots. In these pots were planted healthy adult cladodes of approximate dimensions of 40 × 20 × 2 cm; these cladodes were intentionally infected with this pest.

As observed, in a plastic pot with a diameter approximately of 20 cm, two healthy cladodes were planted with the cladode infected with the cochineal pest placed between them. The spread of the pest is carried out through the points of contact between cladodes, which in this case were the thorns of each specimen, the separation distance between them was 1.5–3 cm approximately. Since the first stage of cochineal pest is mobile, during migration to other parts of the cladode, some cochineals fell to the ground infecting it from the base.

If we consider that in literature it has been reported that during oviposition females deposit about 419 eggs^4^ and we note that in our reproduction model both sides of the infected cladode have about 30 colonies conformed of 1–4 adult cochineal per side, then it is assumed that the spread of the pest towards the healthy cladodes with an infected cladode is enough to contaminate. This was visually verified by observing that during the course of two days both cladodes were already contaminated. In other variants of the reproduction model it was verified that a cladode with the pest is enough to infect four to five adult cladodes approximately of similar dimensions to those used in this study. This reproduction procedure allows us to have several groups of pots where the cochineals are in different maturation stages. The reproduction model proposed in this work is similar to the previously reported reproduction methods^14,15,28,29^ where the planting of the cladodes is also done in pots, however, the pest infestation is carried out in a different way from the one proposed in this work.

**Figure 7:**
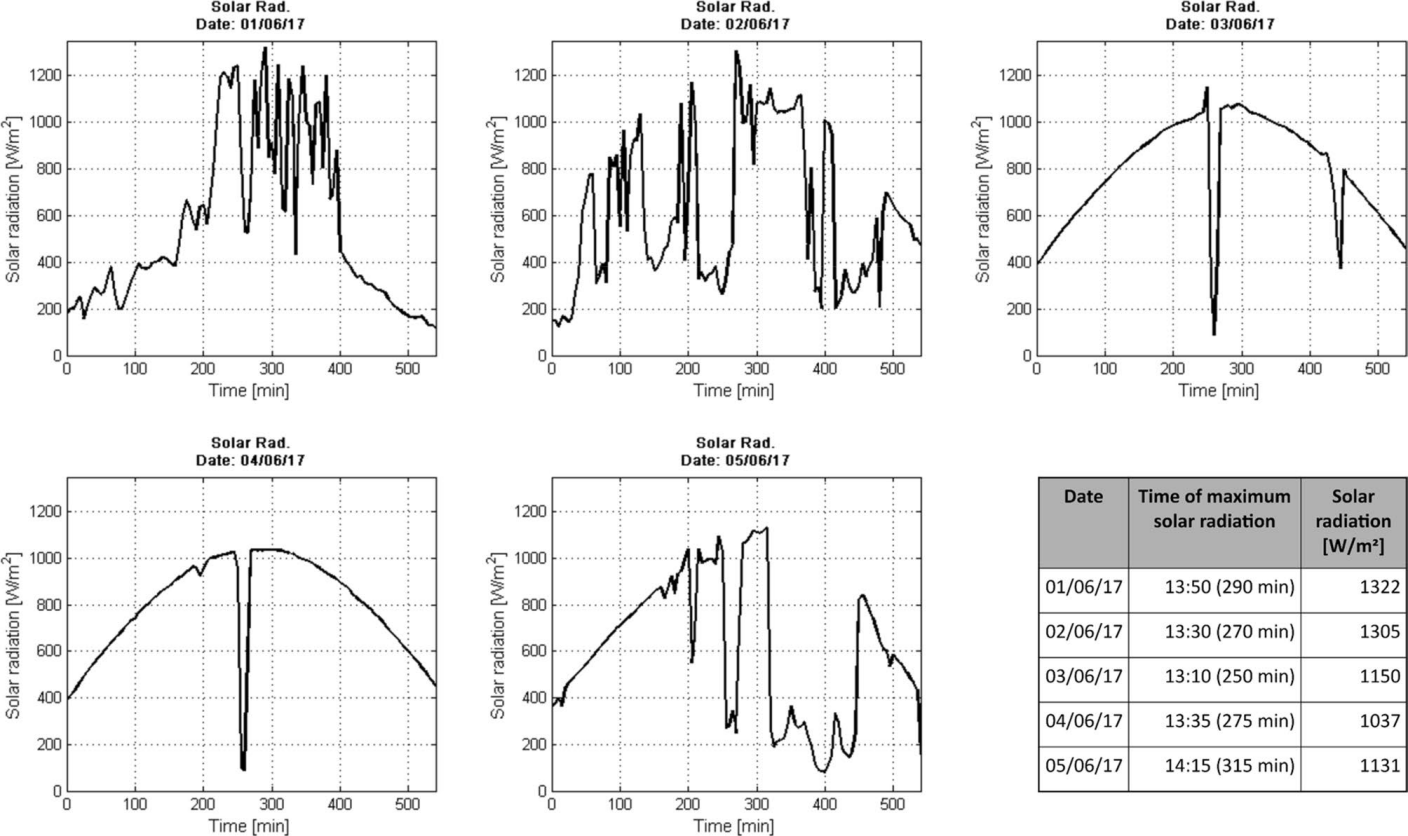
Solar radiation as a function of time (09:00–18:00 h) of the last five days of photoperiod and life cycle of the cochineal. The maximum solar radiation was between 13:00 and 14:20 h, recording a maximum solar radiation of 1037 to 1322 W/m^2^.

In order for the pest to maintain an appropriate lifecycle, the pots are taken out the laboratory to expose the cladodes to sunlight during the day, taking care to avoid the rain and predators that could put the cochineals at risk. In the evening, approximately at 6 o’clock, the pots are returned to their resting place in the laboratory for their care, conservation and adequate control for this study. To be specific, in this study a photoperiod of 9 ± 1 h of direct solar radiation was stablished, to later leave the pots inside the laboratory in absolute darkness, until the next day. This photoperiod was applied for 3 months, for covering the life cycle of the cochineals (90 days approx.). A script development in MATLAB R2014a (The MathWorks, Inc.) software was performed to process the solar radiation detected by the UPT weather station (Vantage Pro2, Davis Instruments Corp.), as shown in Fig. 7, during the last five days of photoperiod from June 01st to 05th, which correspond also to the end of life cycle of the cochineal.

Note that the photoperiod was established in the time range of 09:00–18:00 h, (0–540 min in graphs), corresponding to the time in which the reproduction model was exposed to direct sunlight, so that at the end of the day was placed again inside the laboratory. Figure 7 shows the following data: date, time of maximum solar radiation, also in minutes, and the value of solar radiation [W/m^2^], corresponding to each graph of this figure.

**Figure 8:**
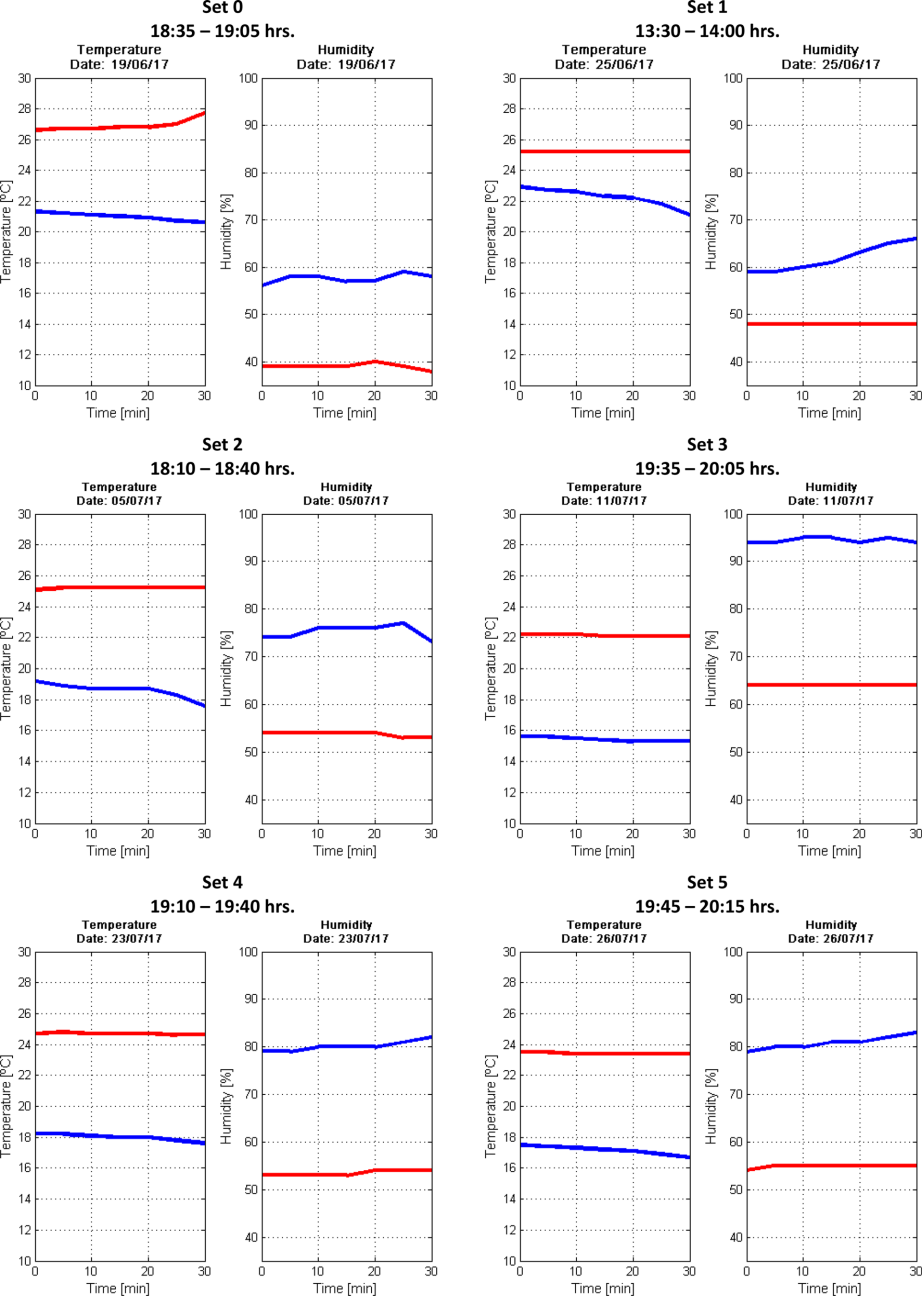
Temperature and humidity (external and internal) registered by the UPT weather station (Vantage Pro2, Davis Instruments Corp.) during the experimental fluorescence measurements for each cochineal set.

The values of temperature and humidity, external (blue line) and internal (red line), recorded by the UPT weather station during the fluorescence spectra acquisition period for each set presented in this work, were processed in a script developed in MATLAB R2014a (The MathWorks, Inc.) software. In Fig. 8 are shown temperature and humidity as a function of time in a temporal range of 35 min, approximately.

The measurement periods of each cochineal set, comprise around 35 min, where most of the cases were performed from 18:10 to 20:15 h. In this figure, data of temperature and humidity recorded by the UPT weather station were plotted, both external and internal with respect to the CIDETyP (Centro de Investigación, Desarrollo Tecnológico, Transferencia de Tecnología y Posgrado) building where the Biomedical Optics Laboratory is located. In Table 3 the temperature and humidity related to six replicates of our experiment are shown, since the cochineal life cycle (approximately 90 days) to the spectral measurement of the cochineal sets (set 0–5). These data are arranged by stage, date, temperature and humidity. These two lasts are in turn divided into the information collected from an online weather service page (WeatherOnline Online Services, www.weatheronline.mx) and by the UPT weather station (Vantage Pro2, Davis Instruments Corp., www.davisinstruments.com). The values, corresponding to temperature and humidity recorded by the UPT weather station, are the result of an average of the data plotted in Fig. 8.

Observe that, in Table 3 exist differences between the temperature and humidity provided by the online weather service page and the UPT weather station. In this regard, since the data from the UPT weather station contain local information of the environment where both the growth of the pest in the reproduction model and the experimental fluorescence measurements were made, these values of temperature and humidity have been adopted in this work.

### Selection of the samples to study spectrally

For this study 3 live-female cochineals of different sizes were selected, of around 480 µm for the small cochineal, 540 µm for the medium cochineal and 790 µm for the largest cochineal. These sizes were determined with a script developed in MATLAB R2014a (The MathWorks, Inc.) software reported in the work of Cárdenas^23^. We assumed that the cochineals are infesting the cladode, in other words feeding from the sap, given their morphological features and size observed and compared with those referred in the available literature^4^.

### Sample preparation

**Figure 9:**
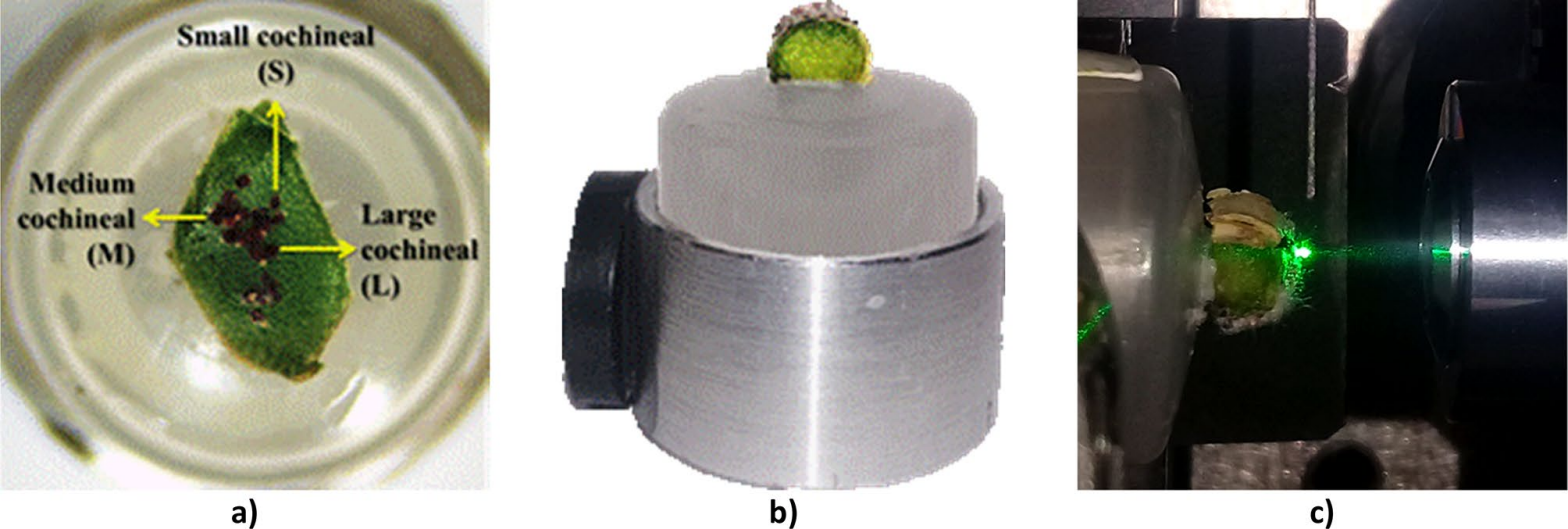
Sample. (**a**) Top view of the biological sample composed by a portion of cladode with presence of female cochineals classified in sizes S, M and L, indicated by yellow arrows. (**b**) Homemade sample-holder for the in-vivo measurement of fluorescence spectra from cochineals in their natural habitat, composed by a cylindrical plastic base where the biological sample is adhered on its top surface and an aluminum ring with two disk-shaped magnets for immobilization purposes. (**c**) Picture taken during the in-vivo fluorescence study of the cochineals in their natural habitat, when the cladode portion with the cochineals in nymph I and II stages is fitted on the sample-holder of the experimental setup.

In order to prepare the biological samples to be studied spectrally, a segment of approximately 6 mm long was cut from one of the infected cladodes planted in the laboratory. The three cochineals previously classified in different sizes were found in this portion as shown in Fig. 9a, where, according to their lifecycle, they are in the stage of nymph I (crawler) and II, that is, in their early growth stages with 23 days old. This cladode portion was fixed on the round base of a plastic cylinder with a glue stick (Lápiz adhesivo DIXON, 36 g). The other end of the round base was inserted inside of an aluminum ring to which two magnets were attached with adhesive (Kola Loka Brocha, 5 GRS) to the sides with 90° in respect to the other, which allows for the proper placement of the sample in the pinhole mount of the magnetic positioning device during fluorescence measurements. Figure 9b shows the lateral view of the sample-holder, where it can be seen that the plastic round base and the sample protrude from the aluminum ring. This device allows us to keep considerable distances and displacements between the sample and the 10 × objective microscope lens during fluorescence measurements as shown in Fig. 9c.

### Measuring procedure of the fluorescence spectra

The in-vivo fluorescence spectra measurements from the cochineals were performed using the optical setup described at the beginning of this section under laboratory conditions of complete darkness, at a temperature range from 20 to 25 °C as well as a relative humidity range from 42 to 80%. Additionally, it was necessary to turn on the excitation laser 30 min before the measurements began in order to achieve a stable fluorescence signal. During this time the output of the laser was obstructed with a dark piece of cardboard to avoid photodegradation of the sample, which was already placed in the sample-holder. Subsequently, the sample was approached to the edge of a dark colored, 374 µm thin plastic sheet, located just at the focal distance of the objective lens. Knowing the thickness of this sheet allows for the sample to be placed at the focal distance of the microscope objective once the plastic sheet is removed by moving properly the sample with the micrometric mechanism along the z-axis. This guarantees that recorded spectra of the cochineal are carried out “correctly”, that is, when the excitation focal point is on the highest part of the surface of this insect.

Once in this location, 10 fluorescence spectra were acquired from each one of the cochineals selected and located in the cladode portion. Three displacements of the sample holder were performed on the z-axis, moving the sample away from and then back toward to the focus plane of the laser excitation beam. Finally, these fluorescence spectra were averaged using a script development in MATLAB R2014a (The MathWorks, Inc.) software, obtaining a smoothed average spectrum of each cochineal as well as the standard deviation between them.

Figure 9c shows a lateral view of the way in which the fluorescence measurements of the in-vivo cochineals were performed. This view, of the interaction area of the excitation beam with a cochineal, allow us to observe that the spot size of the excitation laser is wide enough to cover completely the body of the cochineal.

### Statistical analysis

**Figure 10:**
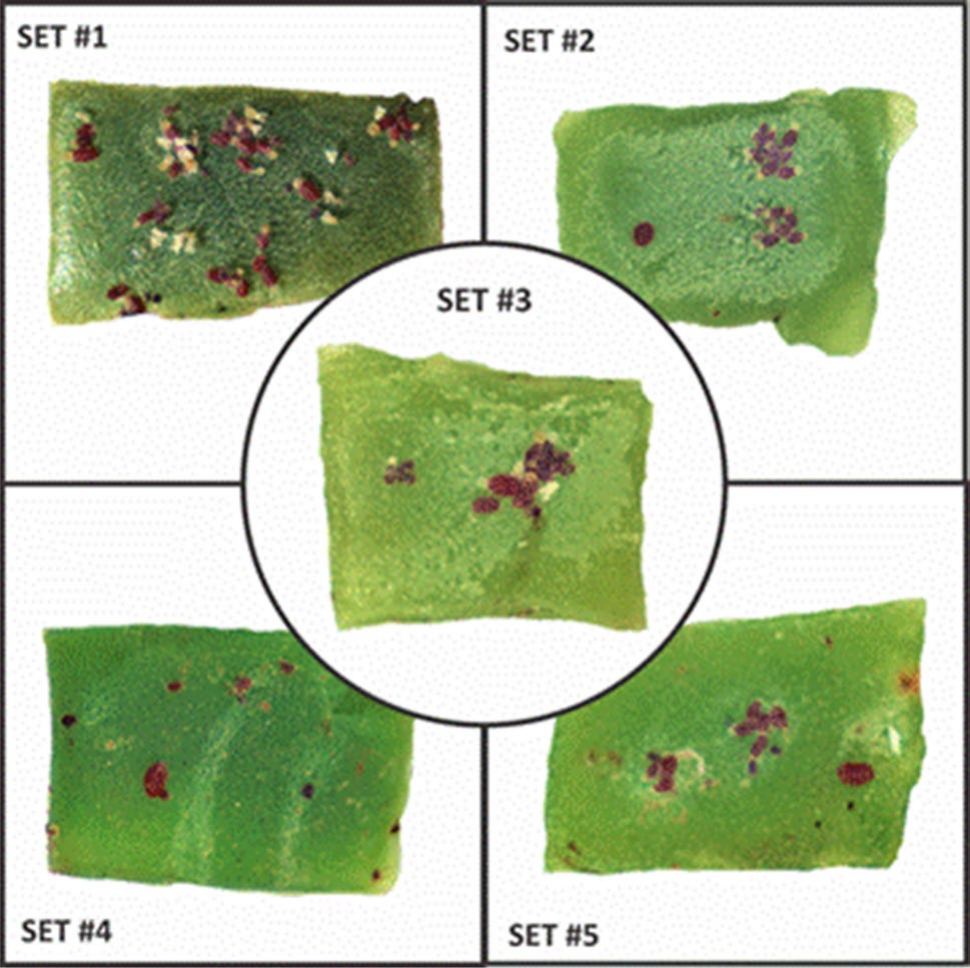
Picture of the five biological samples used for replication of the primary optical experiment of this research work: in-vivo measurement of the fluorescence spectrum of *Dactylopius opuntiae* in fresh cladode segments.

Ten fluorescence spectra were acquired from each cochineal of different size that was present in the biological samples. These spectra were processed with a script developed by the authors in MATLAB R2014a (The MathWorks, Inc.) software, obtaining a smoothed average spectrum of each cochineal as well as its standard deviation. Figures for comparative fluorescence spectra of cochineals of different sizes show average values of fluorescence intensity and its standard deviation (errors bars). Control of reference fluorescence spectra of the background medium (cladode) and a homemade fluorescence standard were taken in all the experiments. Experiments were replicated in five sets of biological samples containing cochineals of similar sizes as is shown in Fig. 10 (see Table 1, which is complementary to this figure below). Statistical analysis of the fluorescence intensity at four wavelengths namely 620, 640, 660 nm and 760 nm were carried out using ANOVA test for the three cochineal sizes (small, medium and large) in each of the five available samples. The statistical values were computed using the Minitab 16 (Minitab, LLC.) software, where the computed *p* values < 0.05 were considered to be statistically significant.

On the other hand, in order to support the comparison between fluorescence spectra mentioned in this work, we have processed and collected spectral information (see Table 2) from the different sets presented in Table 1. This information includes data about the spectral bandwidth, maximum emission wavelength and maximum fluorescence intensity of each cochineal classified in sizes of S, M and L that incorporates the repeatability sets presented above. These values correspond to those obtained with the MATLAB R2014a script which computes the fluorescence spectra averaged and shown in this work.

In order to support the hypothesis of this work by determining how the cochineal size modifies the intensity of in-vivo fluorescence spectra, we have developed a script of our ownership in MATLAB R2014a (The MathWorks, Inc.) software for achieving to fit this intensity as a depending function of the cochineal size in a linear model as observed in Fig. 3b–f. We have processed the ten spectra measured as indicated in the “measuring procedure of the fluorescence spectra” section, emitted by each cochineal from each set presented in Fig. 10, which the values of fluorescence intensity were selected at the wavelength of 640 nm. In Fig. 2 it can be seen that, at this wavelength, the spectra show remarkably visible differences in the fluorescence intensity emitted by the cochineal classified in sizes S, M and L, allowing to distinguish which spectrum corresponds to which size of cochineal. We use the MATLAB function "*polyfit*" to fit the averaged fluorescence intensity values in a linear model, obtaining five graphics that show the average fluorescence intensity as a function of the size cochineal. In order to address the quantitative point of view of this study, we have computed the Pearson correlation coefficient (r) for each set of fluorescence spectra by using the MATLAB function “corrcoef”. For statistical purposes, we have processed values of fluorescence intensity at three additionally wavelengths in 620 nm, 660 nm and 760 nm (data not shown), in order to obtain a linear fit and to calculate linear correlation coefficients.

## Data Availability

The datasets generated during and/or analyzed during the current study are available from the corresponding author on reasonable request.
